# BactoGeNIE: a large-scale comparative genome visualization for big displays

**DOI:** 10.1186/1471-2105-16-S11-S6

**Published:** 2015-08-13

**Authors:** Jillian Aurisano, Khairi Reda, Andrew Johnson, Elisabeta G Marai, Jason Leigh

**Affiliations:** 1 Electronic Visualization Laboratory, University of Illinois at Chicago, 60607 Chicago, IL, USA; 2 Argonne National Laboratory, 60439 Lemont, IL, USA; 3 University of Hawai'i at Manoa, 96822 Honolulu, HI, USA

**Keywords:** Comparative Genomics, Large Displays, Visualization

## Abstract

**Background:**

The volume of complete bacterial genome sequence data available to comparative genomics researchers is rapidly increasing. However, visualizations in comparative genomics--which aim to enable analysis tasks across collections of genomes--suffer from visual scalability issues. While large, multi-tiled and high-resolution displays have the potential to address scalability issues, new approaches are needed to take advantage of such environments, in order to enable the effective visual analysis of large genomics datasets.

**Results:**

In this paper, we present Bacterial Gene Neighborhood Investigation Environment, or BactoGeNIE, a novel and visually scalable design for comparative gene neighborhood analysis on large display environments. We evaluate BactoGeNIE through a case study on close to 700 draft *Escherichia coli *genomes, and present lessons learned from our design process.

**Conclusions:**

BactoGeNIE accommodates comparative tasks over substantially larger collections of neighborhoods than existing tools and explicitly addresses visual scalability. Given current trends in data generation, scalable designs of this type may inform visualization design for large-scale comparative research problems in genomics.

## Introduction

Bacterial genomes--the complete set of genes or genetic material present in bacteria--play an important role in several fields, from the study of micro-biomes to drug development. Bacterial genome sequencing, which determines the complete nucleotide sequence in a bacterial strain's DNA, is increasing at rates that exceed Moore's Law [1], particularly since these genomes are relatively small and inexpensive to sequence. These emerging large collections of complete genome sequence data from bacterial strains are changing the landscape of comparative bacterial genomics.

Comparative genomics is broadly concerned with comparing genomic features across several genomes, to address questions pertaining to evolution and explain variations in different organisms. In particular, *comparative gene neighborhood analysis *involves comparisons across large collections of bacterial genomes to identify variations in neighborhoods around genes of interest. This comparison helps the domain experts generate hypotheses regarding gene function, which is particularly helpful when studying novel or uncharacterized genes [2]. In addition, such comparisons are valuable when identifying the source of differences between related bacterial strains or studying bacterial strain evolution.

Automated methods play a central role in comparative genomics. However, neighborhood-based outliers and common features are difficult to identify through automated methods alone. Visualization can help in this direction.

A variety of visualization applications and techniques exist for genomic data, including tools to support comparative analysis [3]. However, existing techniques are largely not designed to accommodate comparative tasks across large collections of complete genome sequences. In particular, no visual tools exist for comparing gene neighborhoods that scale beyond small stretches of genes in 2-9 genomes. Even if the approaches in existing tools could be scaled to larger collections of genomes, our domain experts found that the fundamental designs did not scale visually or perceptually to allow for large-scale comparative tasks. This scalability issue limits analysis through visualization in comparative bacterial genomics, in particular in the case of comparative tasks across large collections (dozens to thousands) of bacterial genome sequences.

At the same time, novel high-resolution displays have become increasingly adopted in the genomics community, both in the form of personal workspaces featuring multiple monitors, and in the form of collaborative tiled-display walls. While an increase in resolution and display size has the potential to address some scalability challenges, novel visual abstractions are necessary to take advantage of the unique properties of these environments, while avoiding visual clutter.

**Figure 1:**
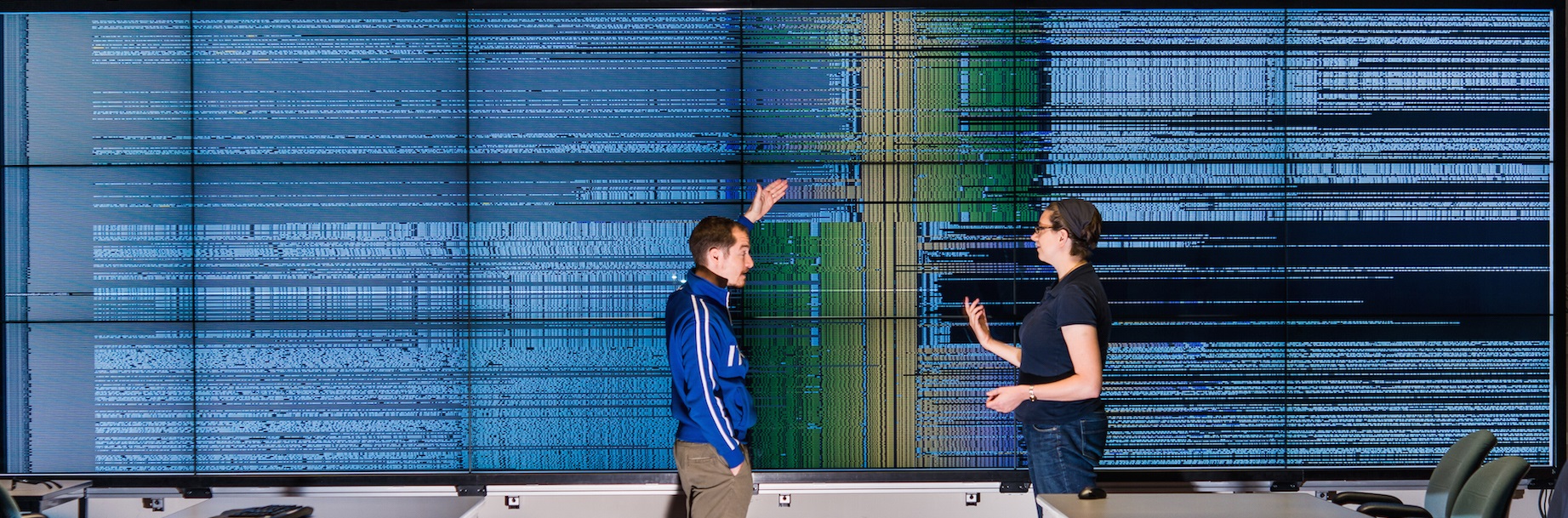
**BactoGeNIE enables comparisons across large collections of gene neighborhoods on large, high-resolution environments**. The visual encodings and interactions are designed to enable data exploration and browsing to enable users to locate and compare neighborhoods of interest and identify features and outliers in content, order and context within these regions. This image shows the neighborhood around a hypothetical protein in all draft *Escherichia coli* genomes from the PubMed database.

**Overview and contributions: **In this work, we introduce a novel visualization approach and application called BactoGeNIE (Figure 1), which stands for Bacterial Gene Neighborhood Investigation Environment. This environment is specifically designed for researchers investigating gene neighborhoods around target genes of interest in large collections of complete bacterial genome sequences, on high-resolution and large display environments. BactoGeNIE was developed over a two year close collaboration with a team of genomics researchers in an industrial research lab setting.

Our contributions include: 1) a data and task analysis for the domain of comparative bacterial genomics; 2) a description of the design process of BactoGeNIE, including a discussion of perceptual issues as well as opportunities and design limitations arising from environments at human scale; 3) an implementation of this design and application to large displays; 4) an evaluation of BactoGeNIE on a case study and with domain experts; and 5) a discussion of the lessons learned through this project.

To our best knowledge, this is the first interactive, large-scale comparative gene neighborhood visualization for big displays. While this work addresses a specific domain topic--comparative gene neighborhood analysis--as sequence volumes exceed the capacity of visualization designs for other research problems, our design decisions may inform comparative genomics visualization development more broadly.

## Related work

Many powerful genome visualization approaches have been developed since the first complete genomes were published in the early 2000s. However, few of these approaches discuss visual scalability in presenting their work, likely because genome sequencing data volumes did not require it. For instance, Nielsen et al. review genome visualizations approaches broadly [3], but do not explicitly discuss visual scalability in popular comparative visualization designs.

In our work with genomics researchers we have evaluated existing approaches, and found that many of the common design decisions in existing tools would not scale visually to accommodate contemporary scales of complete genomes sequences. In the following section we describe these prior designs and discuss challenges in scaling these approaches to larger data volumes or higher-resolution and large-scale displays.

### Visualizations for comparing gene neighborhoods

**Orthology-line techniques**. One common design for gene neighborhood comparisons uses a comparative track between two parallel genomes, each of which is laid-out on an independent coordinate system, with lines connecting similar, or orthologous, genes in different genomes [4-8].

While these applications permit comparisons across several gene neighborhoods, they are not designed to address visual scalability and comparisons over large collections of neighborhoods. The visual design could be 'scaled-up' to accommodate larger data volumes, but orthology lines at this scale become difficult to trace, and visual clutter from many crossing orthology lines limits analysis. In addition, as display size increases, the user needs to trace lines over larger areas, which adds to their cognitive burden.

**Color and layout techniques**. In an alternative approach to depicting orthology between genes in different genomes, similarity or 'orthology' between genes in different genomes is encoded with either color or spatial positioning, or a combination of the two [9,10]. However, these approaches only accommodate comparisons between a few genomes (3-4 in published examples).

SequenceSurveyor is an overview visualization for large-scale genome alignment data that uses color and positioning to encode comparative information about coding sequences within genomes [11]. However, this application is designed to provide overviews of comparative data, and does not emphasize identification of specific comparative features within individual genomes, which is the goal of our work.

### Large, high-resolution displays

Evidence suggests that users take advantage of increased resolution on large displays, and are able to scale-up their perceptual processing to perform visual queries over larger volumes of data [12], with potential benefits for insight formation and discovery in visual exploration [13]. The display scale and resolution permit users to explore a large dataset through physical navigation instead of virtual navigation, allowing visualization designers to exploit *embodied cognition*, such as spatial memory, in complex data analysis scenarios [14-17]. [18] describe applications of such environments to visualizing large, heterogeneous scientific datasets.

These ideas have been applied to comparative genomics by Ruddle et al., who scaled genomics visualizations to large displays. However, many of the designs that were 'scaled-up' to big displays did not require adaptation, because they did not seek to enable the performance of comparative tasks across more genomes. Ruddle et al. also implemented a custom application for large and high-resolution displays, Orchestral, which visualizes--via color and alignment-- copy-number variations across one hundred genomes [19]. Orchestral was designed to enable a different comparative task than BactoGeNIE, and thus features distinct visual encodings and design decisions.

## Methods

BactoGeNIE was developed through a two year long close collaboration with a group of eight comparative genomics researchers, in an industry research lab setting. Several of the researchers specialize in bacterial genomics, and the senior lead of the group has over 20 years of research experience in this field. All researchers in the group have several years of experience conducting research and developing approaches for analyzing genome sequence data. The main focus of the research team was on using computational methods on 'omics' datasets to support research in agriculture.

Over the two year period we conducted weekly meetings. In addition, the lead author was embedded with the team for a period of several weeks, which allowed for daily meetings and observation of the research process with bacterial genomics data.

To gather requirements, we utilized ethnographic observation, interviews and focus groups. As part of this process, we conducted brainstorming sessions and also asked the researchers to describe and critique existing visualization tools, as well as discuss the limitations of automated analysis approaches. We utilized a combination of iterative and parallel design approach, guided by regular feedback. The design stage employed whiteboard sketches, as well as paper and lightweight to fully-developed interactive prototypes.

### Data analysis

**Genomics and bacterial terminology ***Comparative genomics *involves the analysis of similarity and dissimilarity in sequenced genomes. This similarity analysis can occur at several levels of detail, from whole genome comparison to gene sequence comparisons.

Our approach focused on genome sequence data collected from closely related bacterial species. *Bacteria *are small, single-celled organisms whose *genome *is on a single circular chromosome, and potentially several plasmids. A *genome *is the complete genetic material for an organism, and is composed of a linear sequence of subunits called *nucleotides*. This data is regularly stored in 'fasta' format files. Genomic data also includes *annotations*, such as information related to the *coding sequences *for *genes*. This data is stored in annotation files, often in genome feature file (gff) format.

A *bacterial strain *is a genetic variant, which may be described as related to other variants when it possesses similar properties and has a similar genome sequence.

**Specific data entities **In this project, we focus on comparisons in *gene neighborhoods *around genes of interest. The researchers we worked with consider a gene neighborhood to consist of the 10-30 genes closest to a gene of interest. In particular, the researchers sought to identify *orthologs*, or genes with highly similar sequences within and across distinct genomes. The process of identifying orthologs involves sequence comparison algorithms. For this analysis, two genes are *orthologous *if they are found to have highly similar sequences through these sequence comparison algorithms. A set of genes with highly similar sequences are referred to as an *ortholog cluster*.

Due to known sequencing limitations, drafts of complete genome sequences often are broken into pieces that are difficult to *assemble*, or stitch together. These pieces are referred to as *contigs*. Determining how to assemble these contigs and resolving breaks in the complete sequence is a significant challenge. In addition, genome annotations are sometimes incomplete when genomic data is in a draft state, with missing or incomplete annotations. Identifying and resolving such *annotation errors *is a priority when working with newly sequenced genomes.

The *coding sequence *for a *gene *can be found in the organisms' genome. Like a genome sequence, a gene's coding sequence consists of a sequence of nucleotides which determines the sequence and structure of the protein encoded by the gene. To locate a coding sequence for a gene in the genome, researchers refer to gff annotation files, which list a *start and stop nucleotide position *with the contig, as well as which of the two DNA strands on which the gene is encoded. Often the annotation includes common names for the gene or descriptions that cover known functions for the protein encoded by the gene.

### Task analysis

Overall, the domain experts sought support for hypothesis generation pertaining to gene function, particularly when examining previously uncharacterized genes, and bacterial strain behavior and evolution. To this end, the researchers were interested in comparison across large collections of neighborhoods; and in locating variations within one genome, or a set of genomes, in the context of many genomes.

Researchers noted that the value of this analysis for generating hypotheses depends on comparisons across large collections of neighborhoods, since large scale analysis increases statistical significance of features and outliers in ortholog content within neighborhoods. In addition, large collections of neighborhoods allow researchers to assess the weight of evolutionary selection for the content of a particular neighborhood, and make inferences about differences in bacterial strain behavior under different conditions.

#### Functional requirements

Broadly, experts sought to compare the neighborhoods around ortholog clusters of interest in order to identify outliers and common features. These features and outliers would be derived from sets of coding-sequence data attributes, such as gene position, size and orientation, across large collections of neighborhoods. Specifically, the researchers wished to compare and identify features and outliers that fall into three broad categories: **1) gene content in a neighborhood; 2) ortholog order and orientation in a neighborhood; **and **3) context for addressing errors in the data**.

The domain experts indicated that their workflow needed to transition between locating, browsing and exploring tasks, for example:

• Unstructured exploration of a pre-filtered set of contigs,

• Browsing in a location of interest, or

• Locating specific genes or ortholog clusters of interest within a complete set of draft bacterial sequence strains.

The researchers further noted that visualization could particularly enable them to move smoothly between locating, browsing and exploring tasks, where automated approaches tended to require a predetermined target and/or location with little room for exploration.

Below we identify specific configurations of coding sequence data attributes within the neighborhood of an ortholog cluster of interest. These configurations inform the design of BactoGeNIE. The figures provided in this section are derived from paper prototypes we developed in collaboration with the domain experts.

**Gene content in a neighborhood**. Variations in the content of a neighborhood around an ortholog cluster of interest can include the presence or absence of an ortholog in a neighborhood or set of neighborhoods. These variations arise from insertion or deletion events in bacterial strain evolution. Since bacterial genomes often cluster genes involved in similar functions within the genome, changes in neighbors around a gene of interest may signal changes in function, such as an alteration in a biochemical pathway.

**Figure 2:**
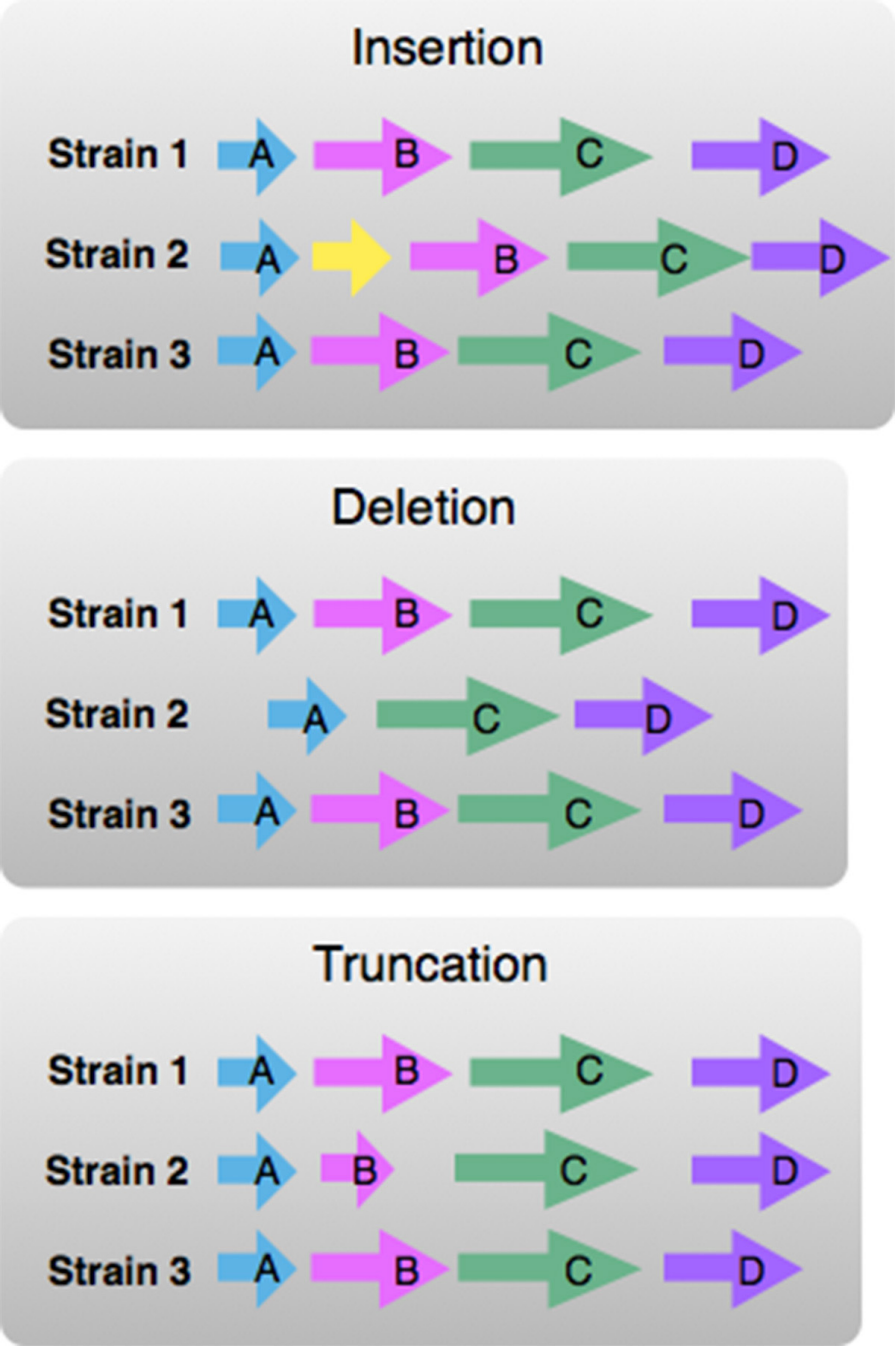
**Content variations: Insertion, deletion and trunction**. In this prototype visual encoding, each horizontal line represents a portion of a neighborhood around orthologs, in 3 bacterial strains. Orthologs have the same color and label. The insertion illustration shows a yellow gene in strain 2 whose ortholog is not present in strains 1 and 3. The deletion illustration has gene 'B' missing from strain 2, while it is present in strains 1 and 3. The truncation illustration shows gene B with smaller length in pixels in strain 2, corresponding to a smaller length in nucleotides compared to its orthologs in strains 1 and 3.

In addition, a variation in a gene's size, in terms of its length in nucleotides, indicates a potential truncated gene. Since a gene's sequence encodes a protein sequence, whose 3 dimensional structure and function depends on this sequence, a significant change in length indicates a significant change in gene-product function. These variations are depicted in Figure 2.

**Figure 3:**
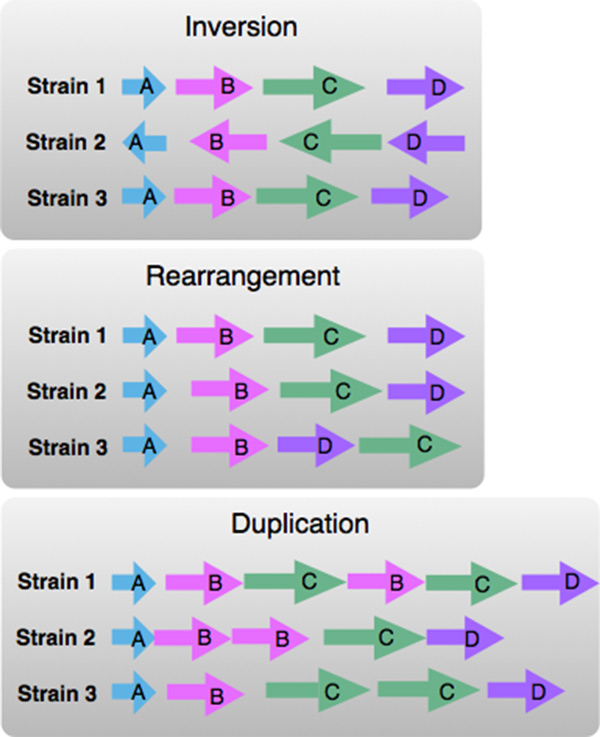
**Order variations: inversion, rearrangement and duplication**. In this prototype visual encoding, each horizontal line represents a portion of a neighborhood around orthologs, in 3 bacterial strains. Orthologs have the same color and label. The inversion illustration shows orthologs in strain 2 with a different orientation, when compared to strains 1 and 3. The rearrangement illustration shows orthologs in strain 2 in a different order, compared to strains 1 and 3. The duplication illustration shows strain 2 with two copies of gene 'B' in strain 2, and two copies of genes 'B' and 'B' in strain 1.

**Ortholog order and orientation**. The domain experts wished to identify trends and outliers in ortholog order within large collections of neighborhoods around ortholog clusters of interest. These neighborhoods may have identical gene content, but significant differences in the order, number or orientation of orthologs, for instance from rearrangement, duplication or inversion events, depicted in Figure 3. Such differences are significant because variations in order may impact gene expression--because sets of gene neighbors are often transcribed in tandem in bacteria.

**Context for addressing errors in the data**. In addition to presenting attributes pertaining to content, order and orientation, researchers introduced data verification tasks that also required coding sequence attributes related to genomic context. This type of task was noted by researchers as a high priority, because data verification could be significantly strengthened by visualization.

**Figure 4:**
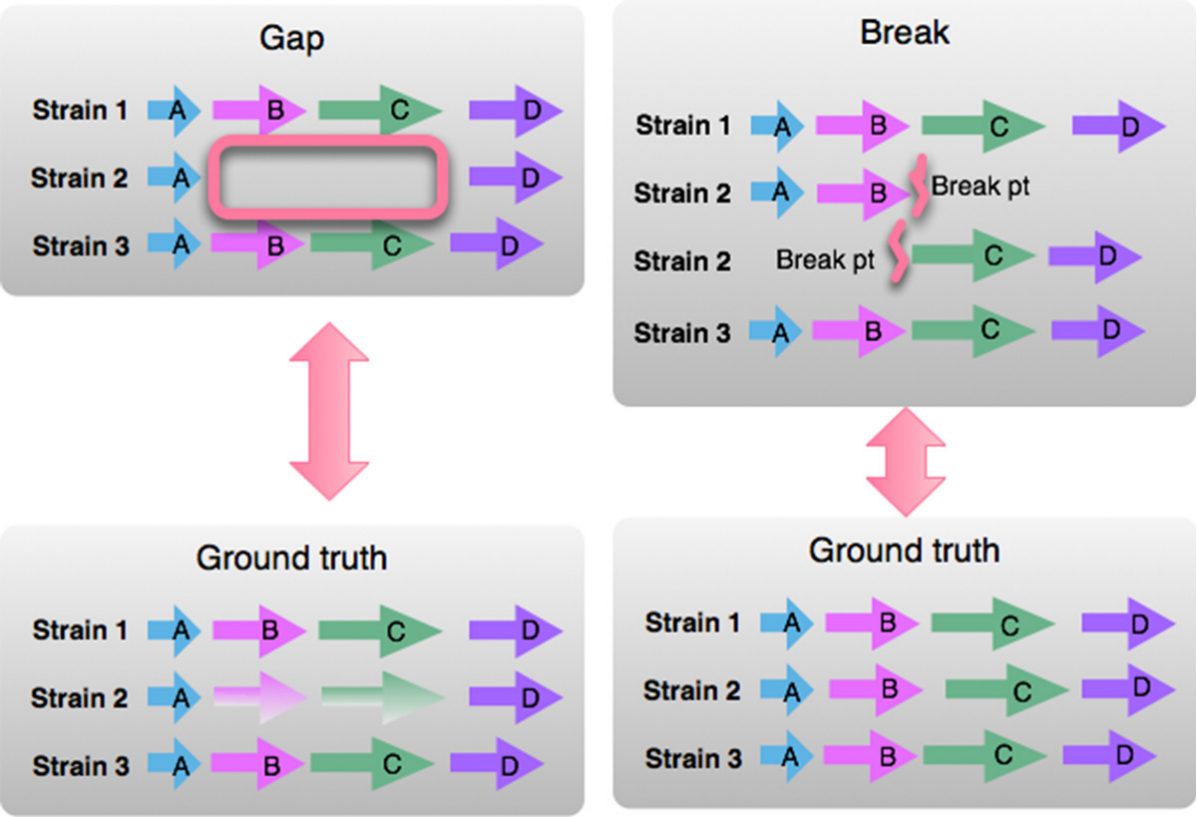
**Context variations: gaps and breaks in assembly**. In this prototype visual encoding, each horizontal line represents a portion of a neighborhood around orthologs, in 3 bacterial strains. Orthologs have the same color and label. These context variations in neighborhoods indicate potential errors in data generation. The first, shows a gaps in strain 2, highlighted with a pink box, when compared to strains 1 and 3, indicating a potential errors in genome data generation. The second, illustrates breaks in genome assembly, and shows how comparative views may help users resolve such breaks.

A variety of errors can arise in the process of generating complete genome sequences. Errors in annotation, which will appear as gaps between otherwise tightly clustered genes, and breaks in assembly, resulting in collections of contigs that would otherwise form a continuous sequence, complicate automated analysis of commonly recurring sequences around ortholog clusters of interest. These features are depicted in Figure 4.

By presenting neighborhoods around ortholog clusters of interest, the researchers believed they could identify potential errors in data generation and form hypotheses that may aid in fixing such errors. The strength of the hypothesis depends on the consistency of a feature across many neighborhoods.

#### Operating requirements: big data and high resolutions

In addition to the task-related requirements identified above, we also derived several requirements regarding the operating environment. The domain experts had acquired a multi-panel tiled display with four 46 inch panels at 2048 by 1536 pixels, with the explicit goal of enabling high resolution data visualization and multiuser collaboration. The BactoGeNIE software was also tested on a 18 panel tiled display wall at 21.9 by 6.6 feet and 6144 by 2304 pixels. Also, the domain experts had personal workspaces with two monitors each.

Many tasks performed by users are at the level of a single neighborhood: the researchers are often considering a few dozen genes around a gene of interest. However, the analysis also takes place across large collections of neighborhoods, where it benefits from an increase in vertical pixels: the large display scale allows more neighborhoods to be displayed and compared. There are also instances where researchers noted benefits of an increase in horizontal pixels, for example in the initial stages of browsing and exploring long contigs, or in cases where a subset of the strains have a large sequence inserted within a neighborhood, or where a section of a neighborhood has been duplicated; such sections can appear at potentially considerable distances within a contig, and benefit from a larger horizontal area.

**High-density encoding and layout **Many visualizations for comparing gene neighborhoods were noted to be ill-suited to supporting large-scale comparison tasks. These visual approaches were found to be fundamentally low-density, in that the visualization is not equipped to show information at high-density even when given the pixels to do so. For some encodings, in particular the ones that encoded orthology by drawing lines between coding sequences or showed text labels by default for all genes on display, we estimated that an increase in the number of neighborhoods on display would produce an increase in visual clutter, and impede the performance of analytic tasks. In other cases, the layout adopted to arrange neighborhoods limits the number of contigs that can be shown without distortions, such as circular layouts. The identified requirement was a *high-density visualization *that accommodates spatial compression in order to show data across a high-resolution display without visual clutter.

**Perceptual scalability **In working with domain experts on tiled display walls, as well as on personal multi-monitor display systems, we noted that many designs for comparing across gene neighborhoods do not *scale-up spatially across a big display*, because an increase in display size seemed to hamper the perception of data and relationships. For instance, scaling up some representations to a large display, moved related entities to opposite ends of the display, limiting direct visual comparison through eye movements. As a result, users appear to need to hold targets in working memory and perform a time-intensive visual search across the display, limiting the use of cognitive resources for higher-level operations. In addition, encodings of orthology that cannot be easily perceived at a distance require users to step-up to the display, making it difficult to perceive patterns in the larger dataset.

We noted these problems with orthology-line-based comparative gene neighborhood visualizations, but also with prototypes that did not provide the opportunity to *spatially cluster related neighborhoods*.

### Visual encodings and interaction design

In this section, we relate the data, tasks and requirements derived above to the BactoGeNIE visual encodings. Our top level design consists of a single global view, with details on demand, brushing and linking, and support for grouping and aligning collections of genomes. Since the task requirements captured through interviews, focus groups and discussions with the domain experts focused on identifying features and outliers within a single collection of sequenced genomes, we concentrated on supporting these tasks within a single view.

In this view, each row (6-10 pixels high) in the visualization shows one contig from one strain of bacteria; the bacterial strain is indicated by a text label on the left. The row-height was determined empirically, based on the display size and user feedback. Within each contig, a sequence of arrow-glyphs depict coding sequences; each contig may contain from one to hundreds of coding sequences. For each arrow-glyph corresponding to a coding sequence, the position, size, and orientation of the arrow-glyph corresponds to the position, size and strand of the coding sequence. The default color applied to all coding-sequence glyphs is a neutral desaturated blue. Below we describe in detail our mappings.

#### High-density encoding of genes and contigs

Based on the operating requirements identified during the domain analysis and early prototypes, we sought a high-density design for encoding contigs, codings sequences, and orthology clusters.

To create a high-density view, we considered several data abstractions for coding sequences. We considered abstractions that only encoded position relative to other coding sequences in the contig, for instance by just representing the order of coding sequences. However, this would not capture *gaps *between coding sequences, which are important for the *data verification tasks *described previously. In addition, we considered abstractions that did encode coding sequence length in nucleotides. However, doing so did not allow researchers to identify *truncations*. We also considered abstractions that did not capture the strand which encodes the protein. However, this information is vital for the identification of *inversions*, as described in the previous section.

For coding sequences, we agreed with the experts that it was essential to encode position, strand and size, as well as show clearly a sequence's contig and bacterial strain. We represent coding sequences via the arrow-glyph described earlier. The direction of the arrow encodes both the strand and direction of expression, allowing researchers to identify potential groups of coding sequences that are 'expressed' in tandem, and inversions. Alternative representations of the coding sequence strand that we have explored include offset layouts, where coding sequences on one strand are positioned slightly higher than coding sequences on a different strand within the boundary of the contig. However, spatial positioning within a glyph has been shown to be less effective when depicting many entities on a high resolution display [15]. We also considered alternative glyphs, such as perpendicular lines or lines with a dot; However, the domain experts judged the arrow glyph to be more effective.

Orthology is primarily encoded through color. Instead of showing orthology by drawing lines between orthologous coding sequences, which limits information density and increases visual clutter, we apply a color across all orthologs through a set of selection and brushing and linking interactions. Given the large number of genes on display, we could not apply unique colors to all genes. Instead, the user selections drive the application of color across orthologs. There are several options for the application of color. Brushing and linking highlights in yellow the orthologs on screen. Users can then brush to apply a persistent color of their choice to an ortholog cluster, effectively 'tagging' the orthologs. Alternatively, the user can use an *ortholog-cluster neighborhood targeting function*, which groups contigs containing a selected ortholog cluster, aligns the entire contig to the cluster and applies a color gradient to the orthologs in the neighborhood.

**Figure 5:**
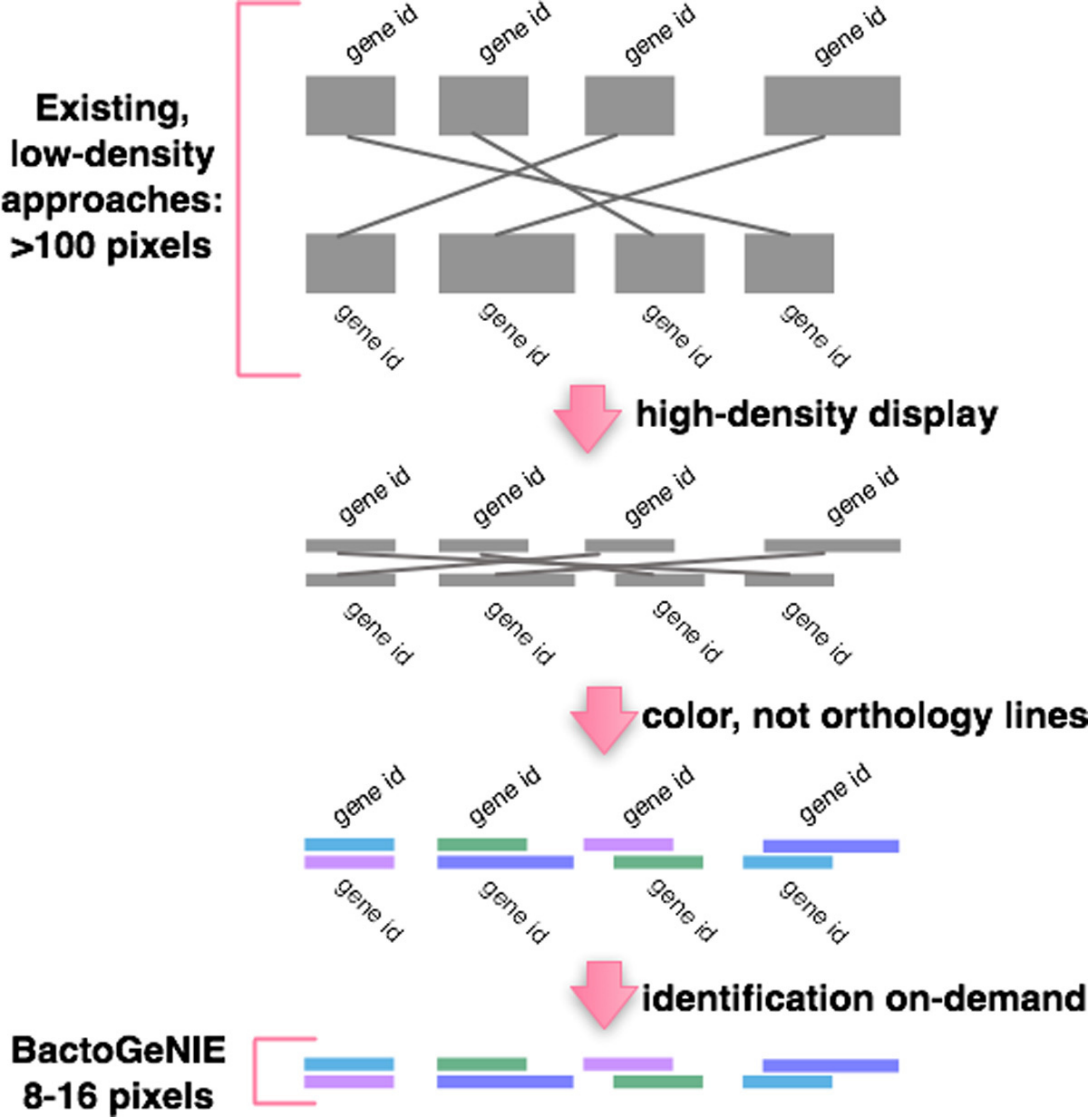
**This diagram illustrates an iterative adaption of an existing 'low-density' design, to the high-density encoding adopted by BactoGeNIE**. At the top, we showed existing 'low-density' orthology-line and text-label encodings. The first transformation, shown by the arrow, reduces the number of pixels for each genome and the gap between genomes, which results in visual clutter. Visual clutter is reduced by replacing lines with color to encode orthology. Finally, by removing text labels BactoGeNIE produces a high-density encoding suitable for large-scale comparative tasks.

We did not show by default textual identification information for coding sequences, since this seemed to, at the same time: 1) limit the number of genomes which we could present, 2) increase visual clutter and 3) not enhance the performance of tasks described previously. Instead, we made this information available on demand. By default, gene identities or other textual data that consume valuable screen space are not displayed, but available on-demand. Contig and bacterial strain labels are available by default on the left side of the display, connected to the contig by a line. These high-density design decisions are illustrated in Figure 5.

#### Encoding for perception over big displays

We designed the BactoGeNIE encodings and interactions so that they enable smooth transitions between exploration, browsing and locating tasks, in the context of comparing and identifying the features and outliers described in the earlier section.

**Genome sorting and ortholog cluster alignment**. Our design adopts layouts for contigs and gene neighborhoods that enable juxtaposition and direct visual comparison, limiting the need for visual search over large numbers of entities on large physical display spaces.

To this end, we adopted spatial clustering techniques, by implementing two grouping operations. First, we enable *genome grouping*, which moves contigs containing an ortholog cluster of interest to the top of the display. Second, we enable *ortholog cluster alignment *to line-up orthologs in distinct genomes against a target gene, producing a vertical stack of related neighborhoods. In addition, these contigs are oriented to the direction of transcription of the selected ortholog, to make inversion events more clear. Grouping and aligning also has the effect of using spatial positioning to encode meaningful information, capitalizing on embodied cognition.

**Figure 6:**
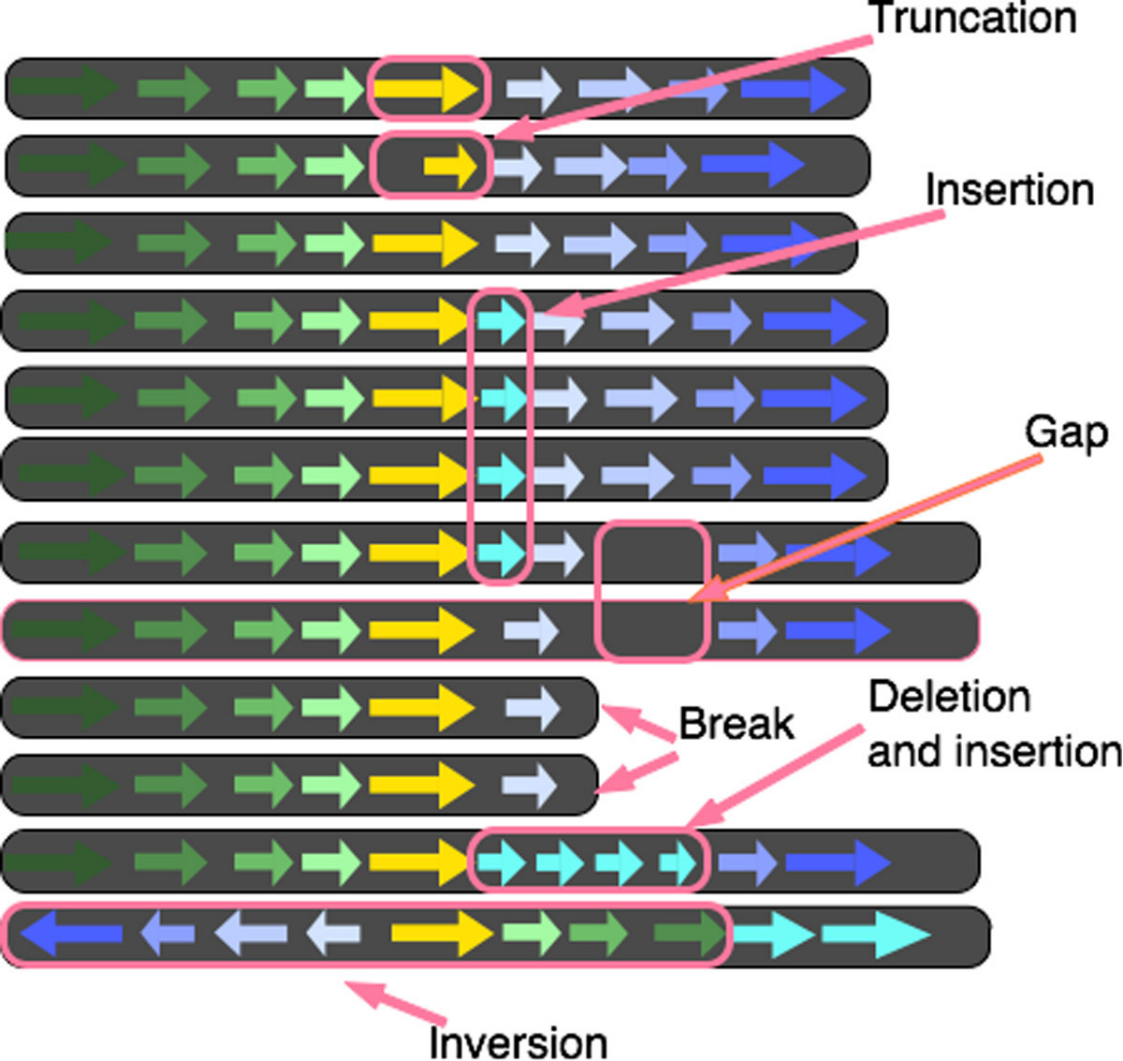
**This prototype visual encoding shows the view following application of the ortholog-cluster neighborhood targeting function to a neighborhood**. Each horizontal line represents a portion of a neighborhood around orthologs, in 3 bacterial strains. Orthologs have the same color. Features and outliers of interest are highlighted in the diagram.

**Ortholog-cluster neighborhood targeting function**. To further enable rapid and immediate comparison and identification of features and outliers described in the previous subsection across large collections of neighborhoods, we developed an ortholog-cluster neighborhood targeting function, the end result of which is depicted in prototype illustration Figure 6, as well as in the application in Figure 1. This function combines spatial clustering, though contig-grouping and alignment against a target gene or ortholog cluster in one contig, as well as coloring on a gradient.

Given a target--an ortholog cluster or gene--ortholog-cluster neighborhood targeting function groups the contigs in order to bring together spatially the neighborhoods that contain the target cluster. We position the target cluster in the center of the display. We then orient the cluster to show the same direction of transcription for the cluster across contigs. Finally, we apply a directional color gradient to the upstream and downstream side of the target cluster. Through the gradient coloring, 30 adjacent genes on that contig are given a color on a gradient, ranging from yellow, for genes closest to the target gene, to either green or blue, for genes farthest from the target gene. Blue genes lie in the direction of transcription, and green genes in the direction opposite to transcription for up to 15 genes on either side of the target gene. The gradient highlights in this manner the order and distance from a selected ortholog cluster target.

From Figure 6, we can see that the end result highlights particular features and variants across sets of neighborhoods, suggesting that this design will make it easier for researchers to identify such variants across large collections of neighborhoods.

**Details on demand: brushing and linking**. Hovering over a gene opens a menu which shows detailed information about that gene and provides options to target that gene for further analysis, including applying a color to the gene and its orthologs, and as well as sorting and aligning contigs and targeting the gene's ortholog cluster. The ability to apply selected colors to an ortholog cluster of interest effectively allows users to tag genes for comparison and identification of features and outliers within the related neighborhoods.

Users are able to navigate through the scene by clicking-and-dragging with a mouse. Vertical movements brings different subsets of contigs onto the display. Lateral mouse movements, shift these contigs right and left, showing different subsets of genes. This type of interaction is important because even with a scalable design not all genes and genomes in a given dataset will be visible. Users can vary the contig height, to control the density of data display. In addition, we provide an 'accordion' action, where the pixel height of a contig is expanded in response to a hover event. The accordion action was requested by users, in order to better enable interaction on the contigs of interest in a high-density layout.

### Implementation

In this project, we used both public and proprietary data. The public genome sequence data was obtained from the PubMed database of bacterial genomic data in draft, or unfinished states. BactoGeNIE was implemented in C++ using the Qt API for graphics and user interface elements. BactoGeNIE requires input of at least two file types, genome feature files, which contain positions of genes and lengths of contigs, and fasta sequence files, which contain raw sequences. The cd-hit algorithm is widely used for comparing protein or nucleotide sequences because it is fast and able to handle very large datasets [20]. After processing, data is stored in a MySQL database, with multiple threads handling data upload and data entry into the database. BactoGeNIE runs both on traditional display environments and tiled-display walls driven by a single machine. The displays used by our biological collaborators were not touch-enabled, and so we designed our system to accept input from a mouse placed close to the display. The researchers used the mouse to interact with the display, and stepped up to the display to discuss points of interest with collaborators. Our design is, however, largely compatible with touch; the only action that would require remapping to touch gestures is the mouse hover event.

## Evaluation and results

Our evaluation approach includes a quantitative analysis of pure scalability in terms of pixels, a qualitative analysis with an in-depth case study, and a qualitative analysis with a group of domain experts.

### Visual scalability evaluation

**Figure 7:**
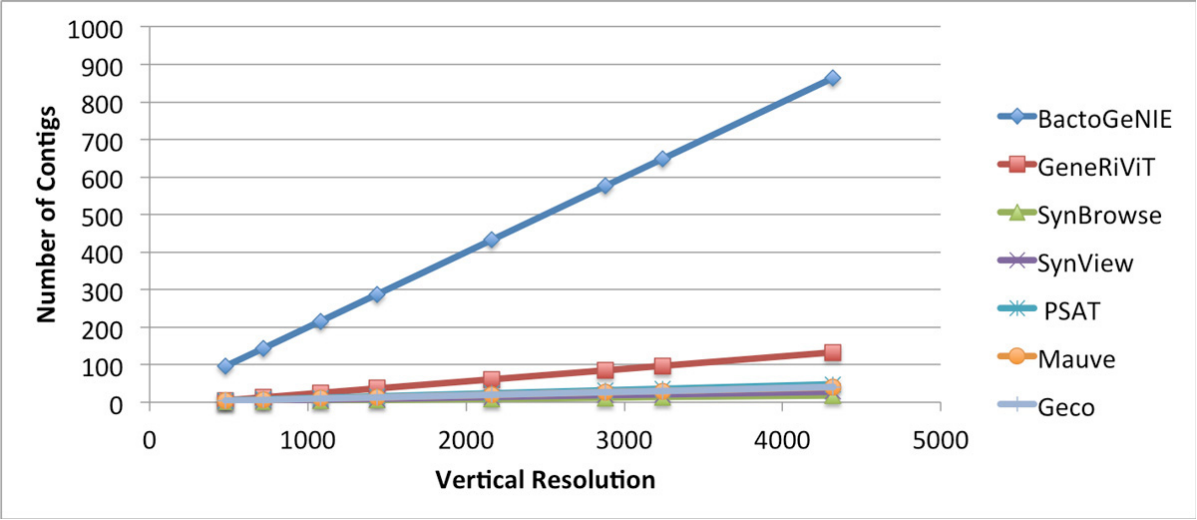
**Large Display Scalability**. Estimated number of contigs that can fit large displays of varied resolutions for related tools. BactoGeNIE is capable of displaying more gene neighborhoods simultaneously than other approaches, and will scale more effectively to large displays.

While the gene neighborhood approaches discussed in the related work section do not explicitly accommodate the upload and visualization of more than a few genomes in one view, we performed a synthetic analysis to measure the number of genomes that each approach could present at various display resolutions. The number of genomes for each tool at varied display resolutions in Figure 7 was computed by estimating the number of vertical pixels used encode two neighborhoods and the orthology between genes in those neighborhoods; we then divided the vertical screen resolution for different displays by this estimate. The result is a quantitative analysis of pure scalability in terms of pixels, given the estimated density of an encoding, and provides a rationale for why many of the approaches in previous work do not scale well to large displays, when compared to BactoGeNIE.

### Case Study: 673 strain *E.coli *analysis

While BactoGeNIE has been adopted as a research tool by the domain experts [21], and has been and is currently employed to investigate genomic data, these data are sensitive and proprietary. The case study reported here reflects a similar usage scenario based on public data; the scenario has been developed in collaboration with the biologists.

In this case study, a biology researcher and co-author of this paper was interested in understanding the function of *Escherichia coli *(*E.coli *) hypothetical proteins. *E.coli *is a common bacteria, which is sometimes pathogenic, and which can also be used in drug design. Hypothetical proteins of *E.coli *have the characteristics of a gene; however, it is not known whether the hypothetical protein is expressed and translated into a protein, or whether this protein product performs a function in the bacteria. Hypothetical proteins are expensive to study experimentally. Early research in the field [2] has indicated, however, that genomic analysis may allow experts to generate hypotheses about the function and importance of this protein, based on observations about its neighbors and the degree of conservation in its neighborhood.

The biology researcher performed her analysis on a large, high-resolution display (21.9 by 6.6 feet and 6144 by 2304 pixels) running BactoGeNIE. She loaded 'draft' or unfinished genomic data from 673 *E.coli *strains from the PubMed database. After examining their visual encoding, the biology researcher performed a set of *exploring, browsing *and *locating *tasks. The biology researcher first *explored *genes in this dataset using brushing and linking. Through these operations she was able to *locate *a coding sequence whose product was a hypothetical protein, and she selected this sequence for further analysis. Next, the biology researcher used the ortholog-cluster neighborhood targeting function to further *locate *all genes which were orthologous to the selected hypothetical protein, across the entire collection of *E.coli *complete genome sequences. Examining the result of the filtering operation, the researcher noted that this hypothetical protein had more than 100 potential orthologs. She then *browsed *through those coding sequences and examined their annotation labels, which presented basic gene information such as a protein name. She noted that this coding sequence was designated as a hypothetical protein within all strains of *E.coli *in the dataset.

**Figure 8:**
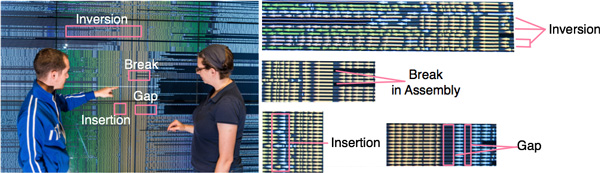
**673 Strain *E.coli *Analysis: Applying the ortholog-cluster neighborhood targeting function to close to 700 strains of *E.coli *produced a view that enabled the identification of features and outliers within the neighborhood of a hypothetical protein, including insertions, inversions and other variations**. In addition, breaks in assembly and gaps between genes indicate potential errors in data generation.

The biology researcher then *browsed *the neighborhoods around these genes, to characterize common features within the neighborhood. In particular, she examined conservation--trying to determine whether the neighborhood around the hypothetical protein was resilient to changes across the genome collection. She identified multiple *insertions, deletions *and *truncations*, as well as *inversions*, as shown in Figure 8. She further *browsed *these variations, applying colors outside the gradient to the variations of interest, and noted *outliers *as well as the strains in which these outliers arose. In addition, she identified a set of common *breaks in assembly *as well as *gaps*, which point to potential *errors in assembly *and *annotation *in the raw data, as described in the Task Analysis section. Several of the neighbors to the hypothetical protein were used in a subsequent round of ortholog-cluster targeting, to study their neighborhood.

The domain experts working in a similar manner on proprietary data developed at this stage hypotheses regarding the function of the examined proteins, based on the annotations associated with common gene neighborhoods and variant features. These hypotheses typically focused on one of two areas: 1) proposing potential processes or pathways in which the target gene may participate or 2) explaining the genetic basis of phenotypic variations within strains with unusual characteristics.

The biology researcher explained that, in the absence of BactoGeNIE, the *E.coli *analysis would have required complex data mining and time-consuming analysis software. The output of these algorithms could be long and difficult to decipher. In addition, the approach would require additional analysis to allow the researcher to identify truncation or inversion events, or relatively uncommon variants. While existing visual tools could help with analysis tasks performed within a single neighborhood in a few genomes, comparative genomics research takes place across large collections of neighborhoods, where it benefits from an increase in display size. Visual encodings and interactions alternative to BactoGeNIE did not scale well and were not attempted. While both small and large displays have been used on these data, the domain experts stated that they found small displays useful only for analyzing smaller, pre-filtered subsets of the data. Overall, in the absence of BactoGeNIE and its high information density encodings, it would have been difficult for genomics researchers to explore or browse this type of large scale, comparative data.

### Domain expert feedback

The domain experts have been given access to both lightweight and fully developed prototypes at regular intervals during the design and development of BactoGeNIE. The eight experts were first shown demonstrations of application usage, and then given the opportunity to use each functional prototype for real analysis scenarios, in the context of their research. We used a "think-aloud" technique when observing the use of the application by the genomics researchers, and also allowed the researchers to use BactoGeNIE independently. Each evaluation session was followed by interviews and discussions to gather feedback. We also observed the use of BactoGeNIE in the context of group meetings, where we noted collaborative hypothesis generation and discussion of observed features and outliers. At each session we collected informal feedback, which was used to drive subsequent development.

This system has been adopted by the domain experts and is now used regularly by the team for analysis of large-scale bacterial genome sequences. Experts noted that BactoGeNIE fits effectively their hypothesis generation workflow, and that it helps them to identify candidates for further computational and automated analysis. The grouping and alignment functions were described as highly useful for enabling comparisons of neighborhoods of interest, especially with large data volumes. The domain experts have further indicated that the visual encodings we used to indicate orthology, both through brushing and linking and the ortholog-cluster neighborhood targeting function, were effective and allowed for rapid identification of variants across large collections of neighborhoods.

Several genomics researchers with years of experience in comparative genomics noted that our approach was unique and that none of the tools they had previously encountered enabled large-scale comparative analysis of gene neighborhoods. One genomics researcher commented that the absence of large-scale comparative visualizations in genomics was a significant technological gap, and that BactoGeNIE helped fill that gap. Other researchers noted that they wished to see this approach extended to large-scale comparative genomics problems for non-bacterial species, suggesting extensibility of the design.

## Discussion and conclusion

The contributions of this work follow the Nested Design Model [22], and span visual encodings, a domain characterization and abstraction into visualization terminology, a description of the design space we explored, an evaluation with domain experts, and a discussion of the merits of our approach.

The domain expert feedback shows that BactoGeNIE implements effectively a design for comparing neighborhoods around ortholog clusters of interest in large collections of bacterial genomes. Further evaluation shows that the environment supports the identification of biologically significant variations and hypothesis generation around gene function and bacterial strain evolution. Overall, the tool allowed the users to incorporate expertise in their data analysis: for instance, users were able to test whether particular strains possess common variants.

As evidenced by the case study, BactoGeNIE accommodates analysis in significantly larger volumes of data, for instance close to 700 strains of *E.coli*, as compared to 9 strains in an application like Mauve. The case study and user feedback suggest that the design is also more visually scalable than other approaches which generally rely on drawing lines between orthologs creating a significant potential for visual clutter.

Our visual encodings and interactions appear to be particularly effective in supporting exploration and browsing for unexpected features and for outliers. For example, gene truncations do not factor in automated approaches which mine for common subsequences, and inversions may be lost unless the mining algorithm takes DNA strands into account. In contrast, truncation and inversion features are easily identifiable in our visualization tool. Furthermore, interactive brushing and linking enabled the domain experts to perform queries on large collections of data.

BactoGeNIE is particularly well suited to large and high resolution environments. Nevertheless, the domain experts also provided feedback which suggested effective use of this tool on personal workspaces and smaller displays, albeit displaying fewer visible neighborhoods at once. Essentially, our design appears to 'scale-down' to smaller displays more effectively than existing designs 'scale-up', suggesting that developing for such environments can also benefit users without access to large display technology. The benefits of embodied cognition in large display environments motivated the design of our alignment, grouping and ortholog cluster targeting encodings. However, embodied cognition was not explicitly investigated in the case study or reflected directly in the user feedback, and constitutes a direction of future work.

In terms of limitations, BactoGeNIE does not take into account the case where coding sequences lie on different strands within the same location. This situation occurs rarely in the datasets evaluated by our collaborators, but would need to be considered in future work. Second, horizontal scalability remains an issue despite the large size of the display. For instance, BactoGeNIE does not provide specific accommodation for contigs with more than one copy of a targeted ortholog cluster. There are circumstances where such genes will be offscreen and not noted by researchers. Finally, the most frequent request from users was to see additional grouping options for contigs following sort and the ortholog-cluster neighborhood targeting function, for instance ones that would group common variants in content, order and context, or apply an ordering that reflects evolutionary distance from phylogenetic trees.

As described in the Nested Design Model [22], at the visual encoding and interaction design level, "the threat is that the chosen design is not effective at communicating the desired abstraction to the person using the system". While our case study does not take the form of a formal user study to validate the visual encoding--partly because of the impracticality of achieving statistically significant results on such a small user group--we nevertheless followed the Nested Model guidelines in validating our encoding approach with respect to known perceptual and cognitive principles, and we further discussed, in context, our encoding choices (see Methods section).

## Conclusion

In conclusion, in this work we introduced BactoGeNIE, a novel visualization design and application that enables comparisons across large collections of gene neighborhoods from complete bacterial genome sequences. BactoGeNIE accommodates comparative tasks over substantially larger collections of neighborhoods than existing tools and explicitly addresses visual scalability. Given current trends in data generation, scalable designs of this type will be increasingly required in comparative genomics research, and we believe the design decisions and guiding principles enumerated here may inform such future work.

## Competing interests

The authors declare that they have no competing interests.

## Authors' contributions

JA conceived and directed the design, implementation, and evaluation of BactoGeNIE. KR assisted with design and research on perception and big display visualization design. AJ and JL provided direction for capturing user requirements, designing for big display environments and evaluating visual scalability. GEM helped characterize the domain in terms of data and tasks, articulate the design decisions, structure the case study and the discussion of results. JA and GEM contributed to and approved the final manuscript.

## Declarations

This work and publication has been supported by grants NSF CAREER IIS-1541277, CNS-0959053 (CAVE2) and OCI-0943559 (SAGE). Special thanks to the Computational Biology team at Monsanto for providing the motivation and drive for this project, as well as for their help in evaluating BactoGeNIE. Any opinions, findings, and conclusions or recommendations expressed in this publication are those of the authors and do not necessarily reflect the views of the funding agencies and companies.

This article has been published as part of *BMC Bioinformatics *Volume 16 Supplement 11, 2015: Proceedings of the 5th Symposium on Biological Data Visualization: Part 1. The full contents of the supplement are available online at http://www.biomedcentral.com/bmcbioinformatics/supplements/16/S11
